# The role of pathogens in diabetes pathogenesis and the potential of immunoproteomics as a diagnostic and prognostic tool

**DOI:** 10.3389/fmicb.2022.1042362

**Published:** 2022-11-14

**Authors:** Muhammad Umar Sohail, Fathima Mashood, Andreas Oberbach, Sareena Chennakkandathil, Frank Schmidt

**Affiliations:** ^1^Proteomics Core, Weill Cornell Medicine, Doha, Qatar; ^2^Experimental Cardiac Surgery LMU Munich, Department of Cardiac Surgery, Ludwig Maximillian University of Munich, Munich, Germany

**Keywords:** diabetes, infections, *Staphylococcus aureus*, early diagnosis, immunoproteomics, biomarkers

## Abstract

Diabetes mellitus (DM) is a group of metabolic diseases marked by hyperglycemia, which increases the risk of systemic infections. DM patients are at greater risk of hospitalization and mortality from bacterial, viral, and fungal infections. Poor glycemic control can result in skin, blood, bone, urinary, gastrointestinal, and respiratory tract infections and recurrent infections. Therefore, the evidence that infections play a critical role in DM progression and the hazard ratio for a person with DM dying from any infection is higher. Early diagnosis and better glycemic control can help prevent infections and improve treatment outcomes. Perhaps, half (49.7%) of the people living with DM are undiagnosed, resulting in a higher frequency of infections induced by the hyperglycemic milieu that favors immune dysfunction. Novel diagnostic and therapeutic markers for glycemic control and infection prevention are desirable. High-throughput blood-based immunoassays that screen infections and hyperglycemia are required to guide timely interventions and efficiently monitor treatment responses. The present review aims to collect information on the most common infections associated with DM, their origin, pathogenesis, and the potential of immunoproteomics assays in the early diagnosis of the infections. While infections are common in DM, their role in glycemic control and disease pathogenesis is poorly described. Nevertheless, more research is required to identify novel diagnostic and prognostic markers to understand DM pathogenesis and management of infections. Precise monitoring of diabetic infections by immunoproteomics may provide novel insights into disease pathogenesis and healthy prognosis.

## Introduction

Diabetes Mellitus (DM) is a complex metabolic disorder characterized by abnormal immune-inflammatory processes that increase vulnerability to severe infections. About 463 million people have DM worldwide, and future projections predict that this figure will grow to 700 million by 2045 (Saeedi et al., 2019). More than 90% of diabetic patients have diabetes mellitus type 2 (DM2), which leads to severe micro-and macro-vascular diseases and places a massive burden on the healthcare system (Chatterjee et al., 2017). People with unmanaged hyperglycemia are at greater risk of experiencing severe medical complications, resulting in lower quality of life, higher medical costs, and mortality (Baena-Díez et al., 2016). Persistent hyperglycemia induces generalized vascular damage, resulting in hypertension, retinopathy, nephropathy, and neuropathy (Chatterjee et al., 2017).

Industrialization, a sedentary lifestyle, smoking, and calorie-rich foods are established risk factors for developing DM2 (Kolb and Martin, 2017). Although these only partially explain the pathophysiology of the disease, chronic infections with environmental or opportunistic pathogens are the other significant contributors to the disease pathogenesis (Tayebi et al., 2021). Pathogens, particularly enteroviruses, *Chlamydia pneumoniae*, herpes simplex virus (HSV), and *Helicobacter pylori*, enhance the risk of cardiovascular events and have the strongest relationship with insulin resistance (Fernández-Real et al., 2006; Nabipour et al., 2006). Blood, skin, urine, and respiratory tract infections are common in diabetic patients that may cause irreversible organ loss or death (Abu-Ashour et al., 2017). According to a meta-analysis of 97 prospective studies involving 820,900 patients, DM-associated infections were one of the leading causes of death (Rao Kondapally Seshasai et al., 2011). Critchley et al. (2018) reported that impaired glycemic control [elevated levels of glycated hemoglobin (HbA1c)] has a positive association with severe infections compared with healthy controls or DM patients with optimal HbA1c. The infection promotes inflammatory markers such as interleukin (IL)-6 and C-reactive protein (CRP) and prevents insulin secretion or interaction with receptors. Furthermore, pathogen’s secretory proteins, such as lipopolysaccharides (LPS), activate toll-like receptors and autoimmune reactions that eventually lead to abnormal energy harvest, obesity, and insulin resistance (Manco et al., 2010). Fernández-Real et al. (2006) observed that higher antibody titers against *C. pneumoniae*, enteroviruses, and HSV are associated with insulin resistance in DM2 patients. Schneider and von Herrath (2014) found that viral infections may act as a longitudinal factor during the induction of autoimmune antibodies against pancreatic β-cells and progression to DM type 1 (DM1). Similarly, other studies also report that infections accelerate the initiation and progression of autoantibodies synthesis at an early stage of the disease and lead to clinical DM1 (Dotta et al., 2007; Morse and Horwitz, 2021). Likewise, DM2 is also associated with infections, including *Staphylococcus aureus*, *Escherichia coli*, *Klebsiella pneumoniae*, *Acinetobacter baumannii*, SARS-CoV-2, HBV, HCV, HHV8, HSV1, and H1N1 virus (Gan, 2013; Leung and Liu, 2019; Lontchi-Yimagou et al., 2021; Carrillo-Larco et al., 2022). Infections with antibiotic-resistant pathogens, such as *S. aureus* and *A. baumannii*, are common in DM2 patients, resulting in poor glycemic control and prognosis (Al-Sultan, 2014; Yan et al., 2022). Leung and Liu (2019) observed a higher mortality rate in DM2 patients infected with carbapenem-resistant *A. baumannii* complex bacteremia.

Early diagnosis and treatment of infections are critical to prevent or postpone secondary DM complications. Kumar et al. (2006) suggested that every hour of delay in antibiotic therapy raises the death risk by 7.6% in patients with sepsis and septic shocks. However, early clinical diagnosis and appropriate antibiotic selection can be difficult when multiple microorganisms are involved. Some of the most common laboratory methods for infection diagnosis include classic microbial culturing, molecular biology techniques, such as targeted PCR or nonspecific high throughput sequencing, and immunoassay. The former two procedures are either very time-consuming or fail to provide a definitive diagnosis and therefore lack clinical relevance in DM management, mainly because many DM-associated infections are polymicrobial (Hitam et al., 2019). Although early diagnosis is vital, classic culturing techniques may take 24 to 72 hto grow the specimen and test drug sensitivity. PCR is a very sensitive and reliable diagnostic tool. However, the clinical application is restricted because of false-positive results and the low throughput of the assay for simultaneous identification of multiple species, drug resistance, and virulence factors (Yang and Rothman, 2004). On the other hand, sequencing requires a large amount of resources and lacks precision (Wajid et al., 2016). MALDI-Biotyping has further improved the diagnosis of pathogens but still cannot distinguish subspecies (Kostrzewa et al., 2019).

Immunoproteomics is a powerful tool to identify immunoreactive molecules and develop candidate vaccines against pathogens. Immunoproteomics combines proteomics for the detection of immunoreactive antigens expressed during infections (Dennehy and McClean, 2012). High throughput immunoproteomics arrays offer a rapid, sensitive, and specific diagnosis of pathogens and their drug resistance profiles, which are otherwise difficult to culture or are multi-species infections. A viral proteome array comprising 646 viral antigens was developed by Bian et al. (2016) to examine the relationship between viral infections and the early onset of DM1. The study observed a trend toward early Epstein–Barr virus (EBV) infection among DM1 patients, suggesting a potential role of EBV in DM1 development. A similar strategy against most common viral, bacterial, and fungal pathogens shall be implemented utilizing comprehensive serum antigen profiles of DM2 patients to predict novel diagnostic and prognostic markers.

## Common sites of infection and prevalent pathogens

The most common sites of infection and their common inhabitant pathogens are discussed in the following sections. However, we highlight that *S. aureus,* a nosocomial infection, is the most common pathogen associated with significant morbidity and mortality in diabetic patients (Smit et al., 2016). The pathogen has a considerable economic and clinical burden, significantly increasing hospital costs associated with methicillin-resistant *S. aureus* (MRSA) infections. Klekotka et al. (2018) reviewed that *S. aureus* is one of the most isolated pathogens in diabetic patients, causing meningitis, sepsis, bacteremia, skin infections, and nasal carriage. Considering *S. aureus* clinical significance, the pathogen will be discussed thoroughly in the DM infection pathogenesis and immunoproteomics sections.

### Skin and soft tissue infections

Skin and soft tissues are the most common infection sites in DM patients, including diabetic foot infections (DFIs) and surgical site infections. In diabetic patients, DFIs account for up to 20% of hospital admissions and 17.4% of hospital-related deaths (Saseedharan et al., 2018; Rastogi et al., 2020). Although DFIs occur on the outer skin layer, bacterial infections can spread the wound to subcutaneous tissues, including fascia, muscles, tendons, bones, and joints. Oh et al. (2018) reported that in DM2 patients, the SSTIs are most frequently caused by *S. aureus*, with a prevalence rate of 50% in DFI. Saseedharan et al. (2018) reported that in DFI patients, *S. aureus* was the most frequently (26.9%) isolated pathogen, followed by *P. aeruginosa* (20.9%). Similarly, Taylor and Napolitano (2004) detected *S*. *aureus* in 35% of postoperative infections (61% of them being surgical site infections) among patients undergoing vascular surgery (40% of whom were DM patients). In DM1 patients, *S. aureus* mostly colonizes the nose and skin (Smith and O’connor, 1966). The bacterium expresses a range of virulence factors, including surface and secreted protein or vesicle. Chronic *S. aureus* superantigens may induce endotoxemia, resulting in systemic inflammation, impaired glucose tolerance, and accelerated DM pathogenesis (Vu et al., 2015). Dunyach-Remy et al. (2016) reviewed global prevalence of Gram-positive cocci (GP), *S*. *aureus*, and MRSA in DFIs, stating that toxinogenic strains of *S. aureus* (secreting exfoliatin, EDIN, PVL or TSST proteins) are often present in infections with a more severe grade and systemic impact. *S. aureus* has been observed to co-exist in many cases with *A. baumannii*, which is also associated with a higher incidence (24.4%) of major amputation in patients with DFI (Cardoso et al., 2017; Ryu et al., 2017).

*Staphylococcus aureus* grows and secretes virulent factors in glucose-rich diabetic conditions, where insulin deficiency prevents or delays immune response (Thurlow et al., 2020). *Staphylococcus aureus* has expanded its glycolytic capacity by acquiring several additional glucose transporters. Carbohydrate transporters in *S. aureus* allow efficient uptake of carbohydrates and support anaerobic growth in inflamed tissues. Eleven carbohydrate transporters have been identified in *S. aureus*, while four of them (glcA, glcB, glcC, and glcU) are strictly responsible for glucose transportation (Vitko et al., 2016). Vitko et al. (2016) observed that in a murine model of wound infection, the inactivation of carbohydrate transporter might reduce glucose uptake and attenuate *S. aureus* growth. Other metabolites, such as fructose, glucose-6-phosphate, and mannose also induced higher expression of *S. aureus* toxins (leukocidins) in DM, indicating a physiologically relevant role of these molecules in DM (Seo et al., 2021). In diabetic skin infections, a lack of nutritional immunity promotes *S. aureus* virulence, including α-hemolysin and a myriad of secreted proteases. Many of these proteins are also expressed in DFI in DM patients, notably the autolysin proteins (Amd), glucosaminidase (Gmd), and the iron-regulated surface determinant proteins (IsdA, IsdB, and IsdH). Therefore, antibody responses against bacterial proteins could predict infection outcomes in patients with *S. aureus* induced SSTIs.

### Respiratory tract infections

Diabetic patients are also more susceptible to various respiratory infections, including *Streptococcus pneumonia, Mycobacterium tuberculosis, S. aureus, Candida albicans*, and influenza virus (Klekotka et al., 2015). Hine et al. (2017) observed a higher prevalence of respiratory infections (23.5%), followed by SSTIs (14.6%) and urogenital tract (10.4%) infections in a large primary care cohort study conducted in the United Kingdom. Hyperglycemia in diabetic patients admitted to an intensive care unit increased the pulmonary bacterial load of *Pseudomonas* spp. (26–32%) *S. aureus* (14–16%) *Klebsiella* spp. (13–14%), *E. coli* (12–13%), and *Enterobacter* spp. (8–12%) (Gill et al., 2016). In The Environmental Determinants of Diabetes in the Young (TEDDY) study, respiratory infections were also positively associated with the onset of β-islet autoimmunity and increased risk of DM1 development (Lönnrot et al., 2017). The nasal cavity is an ecological niche for *S. aureus* carriage (persistent or intermittent) in 20 to 50% of healthy people with a high probability of severe and recurrent infections, particularly in diabetic patients (Wertheim et al., 2005). Furthermore, *S. aureus* carriage rate in adult diabetic patients receiving insulin is 34.0–53.4% compared with the healthy controls (10.7–34.2%) (Sollid et al., 2014). Toxic shock syndrome toxin-1 (TSST-1) and staphylococcal enterotoxin are the most common *S. aureus* superantigens found in the nasal cavity (Klekotka et al., 2018). Schmidt et al. (2017) identified more than 600 *S*. *aureus* proteins in nasal polyps, 115 of which had a strong IgA-and IgG-specific immunoglobulin response.

Many other respiratory pathogens are also associated with DM. The prevalence of pulmonary tuberculosis is 4–5 times higher in diabetic patients than in healthy subjects (Noubiap et al., 2019). Likewise, Badawi and Ryoo (2016) reported a high prevalence rate (54.4%) of Middle East respiratory syndrome coronavirus (MERS-CoV) and H1N1 influenza infection (14.6%) in DM patients. More recent outbreaks of coronavirus disease (COVID-19) also revealed that the disease severity and mortality rates are higher in DM patients (Cyprian et al., 2021). A meta-analysis of 35 studies revealed that DM-related mortality was relatively high (22.14%) in COVID-19 patients (Gupta et al., 2021). Furthermore, DM2 is a known risk factor for severe acute respiratory distress and prolonged viral shedding in critically ill COVID-19 patients (Buetti et al., 2020). Increased susceptibility to pulmonary infections in DM result from reduced activation of lymphocytes, macrophages, and natural killer (NK) cells and reduced immunoglobulins production against antigenic proteins (Lönnrot et al., 2017).

### Urogenital tract infections

DM patients are more likely to get urinary tract infections caused by frequent urination and high glucose levels that provide a suitable growing environment for the pathogens (Woldemariam et al., 2019). High urine glucose concentration provides a good source of nutrients for pathogens to colonize, replicate, and make a UTIs base. Several immune and neuroendocrine defects in DM patients, including reduced T-cell activation, impaired neutrophil function, and low levels of thromboxane B2, prostaglandin E, and leukotriene B4 may lead to a higher UTIs risk (Nabaigwa et al., 2018). *E. coli*, *Klebsiella* spp., *S. aureus*, and *C. albicans* are the most isolated pathogens from UTIs in DM patients (Zubair et al., 2019). Recently, Woldemariam et al. (2019) observed a higher prevalence rate of infections among diabetic patients, where *E. coli* (23.2%), *Coagulase negative Staphylococci* (12.5%), *Enterococcus* spp. (10.7%), *C. albicans* (17.9%) and Non-*albicans Candida* spp. (16.1%) were the most frequently isolated pathogens. Similarly, Nabaigwa et al. (2018) reported that in DM patients, UTIs were positively associated with hyperglycemia with no pathogen detection in the urine of the patients with low fasting blood glucose.

UTIs in diabetic patients are frequently caused by compromised polymorphonuclear leukocyte function, including impaired chemotaxis and phagocytosis (Daryabor et al., 2020). Flagellae of *E. coli* attach to urinary tract epithelium through toll-like receptor 5 and ascend the urinary tract against urine flow (Avalos Vizcarra et al., 2016). Furthermore, adhesin type 1 fimbriae are critical for invading the bladder and evading extracellular antibiotics (Avalos Vizcarra et al., 2016). *E. coli* proteins, such as YbcL, are responsible for delayed immune response in the urinary bladder that helps bacteria multiply easily in the urine and invade the bladder epithelium, a prerequisite for bacterial infection and pathogenesis (Lau et al., 2012). *E. coli* haemolysins act either directly as cytotoxic agents or indirectly by causing inflammation and haematuria (Ambite et al., 2021). Many *E. coli* toxins, such as adhesins, iron acquisition factors, polysaccharide capsules, lipopolysaccharides, and invasins are associated with UTIs and can also spread to the blood and cause systemic infection (Ambite et al., 2021). Floyd et al. (2015) analyzed biofilms of uropathogenic *E. coli*, proposing a novel role of proteomics in uncovering host-pathogen interactions for early diagnosis and treatment of UTIs.

### Blood sepsis

Sepsis and septic shock can arise from an infection anywhere in the body, such as UTIs, pneumonia, or skin wound. Sepsis can be caused by a bloodstream infection leading to fever, septic shock, and a fatal decrease in blood pressure. Diabetic patients have a 4.4 times greater risk of bloodstream infection than non-diabetic patients and are more vulnerable to sepsis of uncertain cause (Stoeckle et al., 2008). Diabetic patients are more likely than non-diabetic to be colonized by MRSA (Stacey et al., 2019). A 25-yearlong study using the National Hospital Discharge Survey US discovered that sepsis occurred in 12.5 million of 930 million acute-care hospitalizations, with DM present in 17% of the sepsis cases (Esper et al., 2009). Moreover, post-sepsis complications and rising mortality are more common in diabetic patients (Frydrych et al., 2017).

Stoeckle et al. (2008) observed that sepsis was more common in diabetic patients than non-diabetic patients, and UTIs were the most common route of sepsis. In general, *E. coli* was the most common etiological agent of sepsis in both diabetic and non-diabetic patients. However, *K*. *pneumoniae* was a more common pathogen in diabetic patients alone. Patra et al. (2017) studied microbial etiology of bacteremia, reporting a high prevalence of *E. coli* (19.7%), *S. aureus* (15%) *Streptococcus* sp. (10.2%), *Staphylococcus sp.* (9.4%), *Enterococcus sp.* (7.84%), and *Klebsiella* (5.5%) in DM2 patients. The infection rate was far higher in uncontrolled DM2 than in controlled DM2. Thaiss et al. (2018) reported a strong association between HbA1c and serum levels of pathogen recognition receptor (PRR) ligands. Bacteria with increased glucose import capacity, such as *S. aureus*, have a better glycolytic flux during infection, enhancing pathogen survival and pathogenicity rate (Vitko et al., 2016). Similarly, *Streptomyces achromogenes* and *P. aeruginosa* infections can also produce irreversible damage to pancreatic β-cells (Schuetz et al., 2011). Chronic bacteremia and prolonged sequestration of viable microorganisms in the liver, lung, kidney, and spleen results in lower IFN-γ and interleukin (IL)-12 cytokine due to fewer antigen–responsive T lymphocytes in diabetic mice (Yamashiro et al., 2005).

### Gastrointestinal (GI) infections

DM increases the risk of enteric infections and intestinal barrier dysfunction, elevating systemic infection risk. The GI tract hosts a complex microbial community, including archaea, bacteria, fungi, protozoans, and viruses, that dynamically influence immune, endocrine, and metabolic homeostasis (Sohail et al., 2019b). Gao et al. (2022) reported that in obese people, bacterial DNA-containing extracellular vesicles (mEVs) could easily cross the gut barrier and deliver microbial DNA into β cells, causing cell injury and impaired insulin secretion by activating cGAS-STING pathway. Several studies have reported changes in the gut microbiome and phage community composition (phageome) in DM2 patients (Sohail et al., 2017, 2019b; Chen et al., 2020). Yao et al. (2018) conducted a meta-analysis of fourteen studies involving 1,841,653 participants and observed that DM significantly increased the risk of enteric infections. Individualized glycemic control, as measured by HbA1c, correlates with systemic transport of gut microbiome secretions (Thaiss et al., 2018). In rodents, infection with systemic *Salmonella* analog, *Citrobacter rodentium*, has been shown to cause hyperglycemia (Thaiss et al., 2018). Chen et al. (2020) observed an increase in the intestinal phage population, particularly *Enterobacteriaceae*, that correlated with a significant increase in systemic LPS concentration in DM2 patients. Furthermore, the authors suggested a consortium of eight phages that could distinguish DM2 patients from healthy controls (ROC-AUC > 0.99).

Several enteric virus infections are also associated with DM pathogenesis. Viral infections are the instigator of gut dysbiosis and disruption of intestinal homeostasis that lead to subclinical enteropathy before DM1 onset (Morse and Horwitz, 2021). Fabiani et al. (2018) found that infection with the hepatitis C virus was associated with an elevated risk of DM2 and vice versa DM2 raised the risk of liver cirrhosis in HCV patients. Changes in enteric virome are linked with several local and systemic disorders, including inflammatory bowel disease, colorectal cancer, obesity, and DM (Oikarinen et al., 2011). Enteroviruses infiltrate gut epithelium for systemic transmission and replication in the immune system, gradually infecting and making pancreatic islet cells vulnerable to autoimmune attack in DM1. Particularly, Oikarinen et al. (2011) observed that infections with enteroviruses triggered systemic inflammation, T-cell infiltration, overexpression of HLA-DR, transglutaminase 2–targeted IgA deposits, and β-cell damage. A bidirectional association between COVID-19 and DM has recently been suggested, with catastrophic consequences in diabetic patients suffering from GIT-form of COVID-19 infection (Cyprian et al., 2021).

### Periodontal disease

Periodontal diseases are common in DM patients and are one of the primary risk factors of DM. Periodontitis is a common complication of DM, which is also associated with other DM complications and exacerbated by uncontrolled hyperglycemia (Nguyen et al., 2020). Similarly, periodontitis is also associated with gestational DM (Abariga and Whitcomb, 2016). Nguyen et al. (2020) conducted a systematic review of the literature to see whether there was a correlation between periodontitis and DM complications. They found that diabetic patients with periodontitis were more likely to develop retinopathy, neuropathy, nephropathy, and cardiovascular complications. Several oral pathogens, including *Aggregatibacter actinomycetemcomitans*, *P. gingivalis*, *Tannerella forsythia*, and *T. denticola*, are associated with hyperglycemia and contribute to periodontal disease development and gingivitis (Feng et al., 2014; Sohail et al., 2019a; Graves et al., 2020). Awan (2020) observed a higher prevalence of *S. aureus* in the dental cavities of diabetic patients compared with the controls. Al-Obaida et al. (2020) observed higher abundances of periodontal pathogens, *A. actinomycetemcomitans* and *Capnocytophaga ochracea*, in the dental cavities of diabetic patients. Bacteria or their products such as LPS from the sub-gingival plaque are thought to trigger the release of pro-inflammatory cytokines (interleukin-1β, interleukin-6, and interleukin-8, and tumor necrosis factor-alpha) that enter the systemic circulation and interfere with insulin signaling, causing insulin antagonism and pancreatic β-cell destruction (Moller, 2000; Chokwiriyachit et al., 2013).

## Pathophysiology of diabetes infection

DM and infections are linked bidirectional, with infections impairing glycemic control and hyperglycemia promoting bacterial growth. Improved glycemic control prevents infection and improves treatment outcomes (Martinez et al., 2017; Ciancio et al., 2018). Pathogens can elicit strong immune responses, effectively activating immune regulatory mechanisms and causing autoimmune reactions that cause β-cell damage or insulin receptor deactivation (Fallahi et al., 2013; Seyyed Mousavi et al., 2017). In diabetic patients, both humoral and cellular immune responses are compromised, resulting in impaired cytokine production and the activation of phagocytic cells. Chronic hyperglycemia continuously stimulates polymorphonuclear lymphocytes and baseline cytokine levels, causing cellular and humoral responses insufficient for pathogens (Dryden et al., 2015). Hyperglycemia impairs neutrophils, significantly reducing their chemotactic migration, phagocytosis, and bactericidal functions (Jafar et al., 2016). Poor glycemic control in DM2 patients also suppresses the immune activity of monocytes and monocyte-derived macrophages (MDM) (Valtierra-Alvarado et al., 2020). Valtierra-Alvarado et al. (2021) observed lower activation markers in MDM from T2D patients in response to an *in vitro M. tuberculosis* infection. Therefore, diabetic patients, especially those with peripheral vascular disease, have immunosuppression, diabetic neuropathy, and impaired blood circulation, making them vulnerable to infections (Berbudi et al., 2020).

Infection-induced IFN-γ downregulates insulin receptor expression resulting in compensatory hyperinsulinemia to keep euglycemia (Šestan et al., 2018). The liver regulates blood glucose levels by glycogenolysis, gluconeogenesis, and glycogenesis. However, in diabetic patients, viral infections jeopardize euglycemia by disrupting glucose homeostasis. Šestan et al. (2018) observed that viral infections activate the inflammasomes, which generate the pro-diabetic cytokine IL-1β that disturbs glucose homeostasis in the liver. Furthermore, viral infections trigger hepatic insulin resistance, accelerating DM pathogenesis. However, not all diabetic patients experience the same risks; improved glycemic regulation is related to lower infection incidence, as shown in many postoperative DM treatment reports (Cancienne et al., 2018a,b). Similarly, in patients with COVID-19 and pre-existing T2D, well-controlled glycemia was related to significantly lower mortality and better disease prognosis (Zhu et al., 2020).

Although hyperglycemia is generally believed as an immune-compromised condition that attracts infections, some research suggests that pathogens may initiate primary tissue insult to exacerbate DM pathogenesis. Since the host’s genetic background plays a relatively minor role in inducing a self-immune reaction, epidemiological and molecular data point to infectious agents as the primary environmental factor that trigger autoimmune disorders (Vu et al., 2015). Adenovirus, cytomegalovirus, enteroviruses, mumps, and rubella are among the viruses linked to the pathogenesis of DM1 (Op de Beeck and Eizirik, 2016). *S. aureus*, one of the most common opportunistic pathogens, has also been linked to the onset of insulin resistance by blocking insulin receptors (Weidenmaier, 2018). Perhaps these infections are a catalyst for initiating a complex chain of immune-inflammatory processes that lead to chronic hyperglycemia (Peleg et al., 2007).

**Figure 1:**
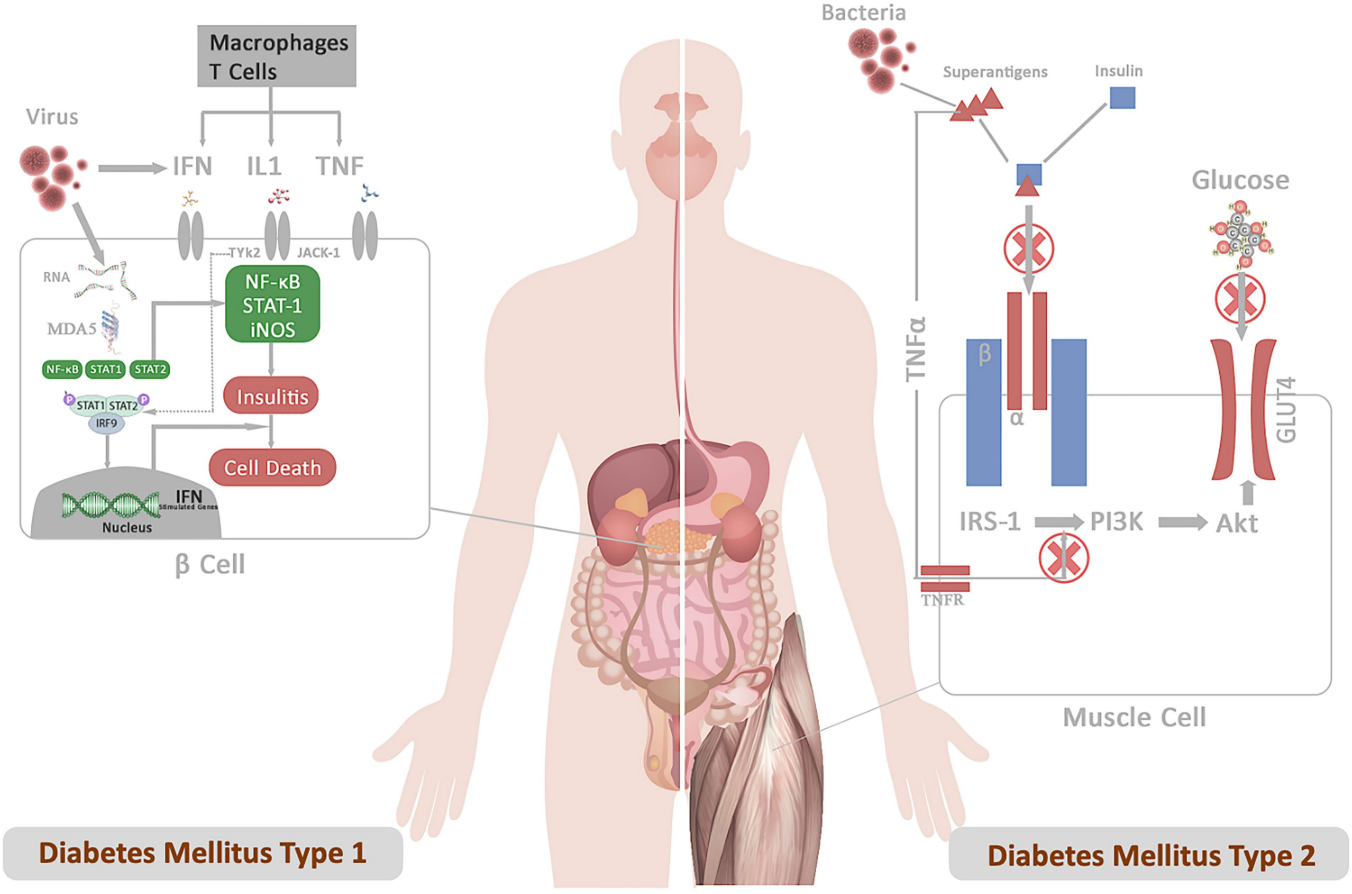
Represents the mechanistic of diabetes infection emphasizing the role of infection in DM1 and DM2 pathogenesis. **(A)** DM1: Following the infection, viral particles release viral RNA in the host cells, which is recognized by the cytoplasmic receptor MDA5. The binding of MDA5 to the RNA activates the transcription factors NF-κB and STATs pathway. The binding of IFN, IL, and TNF to their receptors results in the activation of the JAKs and TYK2 pathway, triggering activation of STAT-1–STAT-2–IFN-regulatory factor 9 complex. These complexes translocate to the nucleus and bind IFN-stimulated response elements in DNA to initiate the transcription of genes involved in host defense. Production of type I interferons contributes to insulitis and cell death. **(B)** DM2: Superantigens secreted from bacterial pathogens (*S. aureus*) (a) prevent insulin binding to the IR and abrogate signal transduction (b) LPS-stimulated inflammatory cytokine block PI3 kinase-Akt pathway. These signals inhibit downstream IR signaling events involved in promoting glucose conversion, reducing recruitment of the glucose transporter GLUT4 to the cell membrane, and impairs glucose uptake.

Several DM1 candidate genes, such as PTPN2, MDA5, and TYK2, also control antiviral responses in both β-cells and the immune system, triggering autoimmune destruction of the cell (Op de Beeck and Eizirik, 2016). Sequence homology between a pathogen-derived peptide and a self-peptide can cause an immune response against self-tissue, activating the release of autoantigens and bystander T-cell activation, resulting in inflammation and cell death (Cusick et al., 2012). The most compelling proof of infection-induced insulin-deficient hyperglycemia and enterovirus infection comes from cases of fulminant DM1, a typical type of DM1 in Japan (Tanaka et al., 2013). Similarly, insulin resistance may occur possibly through *S. aureus* membrane-bound enzyme lipoteichoic acid synthase (LtaS), which inhibits insulin binding to insulin receptors and prevents GLUT4 from being transported (Figure 1; Weidenmaier, 2018). Perhaps these pathogens or their products may not induce DM on their own. Instead, patients are frequently diagnosed with DM during or after an infection, indicating a connection between the two. Furthermore, a greater affinity among diabetic patients for acquiring infections suggests that DM falls in the first order before infection. This indicates that these pathogens or their peptides do not function as the primary trigger but act as confounding factors in impaired insulin production or sensitivity, ultimately exacerbating the disease outcomes.

## Role of virulence factors/proteins in diabetes pathophysiology

Virulence of pathogens and host immune factors may determine the pathogenesis and fatality of DM infection. Pathogen-related factors, such as secretory or surface-attached peptides, play a significant role in adhesion, motility, and invasion while evading host immune responses. Each pathogen can secrete various virulence factors depending on the host environment and infection site. To simplify the role of virulence factors in DM pathophysiology, we focus on *S. aureus* as one of the most common opportunistic pathogens that has a high capacity to cause infections in obese and diabetic patients.

Liew et al. (2015) noted that *S. aureus* infections develop heterogeneous and broadly diverse virulence factors and host immune responses based on their site of infection. Furthermore, in diabetic patients, *S. aureus* needs fewer virulence factors (hemolytic and cytotoxic) to establish severe infection. Particularly, *S. aureus* has increased glucose import capabilities, which aid the bacteria in optimizing glycolytic flux during infection and pathogenesis (Vitko et al., 2016). Even less-virulent *S. aureus* strains survive in higher numbers, producing superantigens and inducing infection in the murine footpad injection model (Tuchscherr et al., 2018). In hyperglycemic environment catabolite control protein A (CcpA) may affect *S. aureus* infectivity, growth, glucose metabolism, and transcription of selected virulence determinants, including the agr locus, altering the transcription patterns of hla, α-hemolysin, and spa (Seidl et al., 2006; Bischoff et al., 2017).

*Staphylococcus aureus* secretes various virulence factors, including lipases, adhesins, and pore-forming toxins (Tuchscherr et al., 2018). Their volumes can increase after the pathogen internalization (Surmann et al., 2018). The secreted toxins, such as increasing adhesins and pore-forming toxins, detour host immunity by enhancing cell attachment or lysis (Thammavongsa et al., 2015).

Toxic shock syndrome toxin-1 (TSST-1), another popular superantigen from *S. aureus*, plays a critical role in DM pathogenesis. Vu et al. (2013) observed that rabbits exposed to *S. aureus* superantigen TSST-1 had impaired glucose tolerance, elevated endotoxin levels, and systemic inflammation. Macrophages and T-lymphocytes are stimulated by TSST-1 to produce inflammatory cytokines. TSST-1 induces a massive release of cytokines that specifically attach to T-lymphocytes’ variable domains, resulting in hyperimmune response clinically characterized as fever, endotoxic shock, rash, and progressive multi-organ failure (Silversides et al., 2010). This nonspecific interaction stimulates less than 20% of the total population of T lymphocytes in diabetic patients, culminating in a severe cytokine storm (Que and Moreillon, 2015).

## Immunoproteomics as novel diagnostic and prognostic tool

### Proteomics

Proteomics and immunoproteomics studies of human body fluids and infection-specific antibodies offer complementary knowledge and hold the potential for developing novel biomarkers for DM infection (Larman et al., 2013; Yu et al., 2022). Proteomics in general has advanced linearly over the last few decades, with two-dimensional polyacrylamide gel electrophoresis (2D-PAGE) and Western blot being the mainstay of comparative proteomics studies. Protein research is crucial for better knowledge of disease pathogenesis and therapeutic response. DM is a polygenic disease with significant contribution from genetic, environmental, and behavioral factors, making it suitable for proteomics research. Most genome-wide association studies (GWAS) of DM identified only a small number of genetic variants contributing to DM risk (Mahajan et al., 2018). A more comprehensive biological readout of these factors can be obtained by proteomics, which analyzes downstream products of gene transcription. Recent technical developments have made it possible to profile circulating proteins quickly and on a much broader scale; a few of these are briefly discussed here.

Proteomics based on 2DE is a powerful tool for simultaneously separating and fractionating thousands of proteins. The technique has significantly evolved to identify and quantify proteins with mass spectrometry or immunological tools. 2DE is used for whole proteome analysis, protein purification and characterization, cell differentiation, drug discovery, disease biomarkers, and bacterial pathogenesis studies (Magdeldin et al., 2014). The technique provides direct visual confirmation of post-translational modifications, which cannot be predicted from the genomic sequence. Proteins of interest can be identified using peptide mass fingerprinting (PMF; Thiede et al., 2005) or antibody probes (Backert et al., 2000). For PMF, the protein of interest is isolated from the gel and digested using Trypsin to produce 8–10 amino acid-long peptides. These peptides can be analyzed with MALDI-TOF mass spectrometer (MS), a fast, sensitive, high throughput, and accurate procedure. Molloy et al. (2001) used 2DE-PMF to identify the outer membrane proteins of *E. coli, S. typhimurium*, and *K. pneumoniae* that are otherwise difficult to identify. Similarly, Kundu et al. (2020) performed 2DE-PMF of PBMCs from comorbid DM-tuberculosis patients, identifying 18 protein spots with differential expression. MALDI-TOF based PMF revealed that these overexpressed proteins are involved in cell structure, cell cycle, signaling, and intermediary metabolism. Kalita et al. (2020) reviewed the application of 2DE-PMF in DM-associated organs and tissues, such as the pancreas (Ahmed et al., 2005), liver, skeletal muscle (Højlund et al., 2008), and adipose tissue secretome (Alvarez-Llamas et al., 2007). Liew et al. (2015) compared host immune response and *S. aureus* exoproteome in SSTI, bacteremia, and healthy carriage using 2-DGE, 2D-immunoblotting, and MS/MS (MS2). Using different proteomics techniques, the authors discovered a diverse exoproteome profile that varied significantly across infection sites. Differential expression of the Isd protein and host cytokine/chemokine response pattern were particularly important in understanding *S. aureus* infection. Luo et al. (2012) combined 2DE, Western blot, and MALDI-TOF MS to discover immunoreactive proteins for *A. baumannii* for vaccine development. Rational proteomics analysis discovered higher anti-OmpA antibody titers in *in vivo* testing of the OmpA antigen in diabetic mice, protecting the mice from intravenous infection with *A. baumannii* (Luo et al., 2012).

While it is true that gel-based techniques were the foundation of the early proteomic research in DM, they frequently have poor sensitivity in finding low-abundant proteins and are time-consuming (Kalita et al., 2020). Parallel advances in sample preparation methods, MS technology, and data analysis have prompted a shift toward gel-free techniques that profile thousands of proteins in a relatively shorter time and with small sample volumes. With higher sensitivity and resolution power, MS uses chromatography for molecules ionization and separation by mass to measure proteins. Chromatography improves the quantitative accuracy and throughput of MS, particularly for analytes with low abundance (Pocsfalvi et al., 2016). There are two types of chromatographic techniques: gas chromatography (GC) and liquid chromatography (LC), each with its own set of specifications and pros and cons. In GC, for example, nonpolar, low-molecular mass, and volatile analytes are vaporized into a mobile gas phase and passed through a liquid layer. LC, on the other hand, is ideally equipped for polar, high-molecular mass, heat-labile molecules. The analytes are dissolved in a liquid mobile phase and passed through a column filled with beads coated with various compounds that serve as the stationary phase.

The precision and sensitivity of the MS can be increased by the depletion of high-abundance plasma proteins and affinity binding techniques. Physical separation of the proteins by liquid chromatography or gel electrophoresis and trypsin digestion prior to MS and follow-up with immunoassays can further improve the diagnostic limits of proteomics (Chen and Gerszten, 2020). Data acquisition techniques in MS also affect the throughput of the analysis. Data dependent acquisition (DDA) MS acquires MS2 data so that the user can define specific precursor ions based on abundance or other criteria. Unlike DDA, data independent acquisition (DIA) acquires MS1 and MS2 data without bias for all precursor ions within isolation windows for full mass ranges without prior knowledge of precursors (Ludwig et al., 2018). Malipatil et al. (2019) quantified insulin growth factor, vitamin D binding proteins, and HbA1C using DIA-MS to predict the effect of diet and exercise on weight loss and insulin sensitivity. Salivary proteomics of diabetic patients identified TXNDC17, ZG16B, and FAM3D as novel biomarkers of hyperglycemia and oral disease in DM2 patients with poor glycemic control (Jia et al., 2021). An MS-DIA based proteomic analysis of infected root canals of DM2 patients identified fortythree overexpressed proteins, the majority of which were related to immune-inflammatory response (Loureiro et al., 2022). Withatanung et al. (2019) performed proteomics of *Burkholderia pseudomallei* infected Neutrophils isolated from DM2 patients observing elevated levels of pro-inflammatory cytokines IL-1β, IL-6, IL-17, and TNFα as well as enhanced apoptosis. Likewise, Gagnaire et al. (2012) used MALDI-TOF MS to detect *S. aureus* delta-toxin in DFI and chronic lung infections. Acar et al. (2022) applied a subtractive proteomics approach to study immunodominant *A. baumannii* exoproteome/surfactome for candidate vaccine discovery. The researchers used IgGs to capture *A. baumannii* proteins and identified them using LC–MS/MS analysis, observing 34 unique proteins and several notable vaccine candidates, including A0A0R4J8QA3, B0V9Z6, B0V885, B0V4F6, B0VD00, B0VAB5, B0V8H0, B0VE52, B0VC68.

Analytical protein arrays or multiplexing have a wide range of applications, including cell signaling pathways and protein interactions analysis, which could aid in the discovery of novel autoantigens for DM diagnosis, prevention, and treatment (Chang et al., 2015). Among many other proteomics platforms, proximity extension assay (PEA) technology based Olink and SOMAScan have attained widespread application in clinical testing and discovering new biomarkers (Liu et al., 2022; Sánchez-Maldonado et al., 2022). SOMAScan is a highly sensitive proteomic multiplexing platform based on affinity capture and low off-rate modified aptamers (SOMAmers). Elhadad et al. (2020) observed over 1,000 proteins in DM2 patients, revealing 24 replicated proteins, of which 8 are novel. Aminoacylase-1, GHR, insulin-like growth factor-binding protein 2, cathepsin Z, and rennin were associated with the incidence and prevalence of DM2. Aminoacylase-1 was associated with both prevalent and incident DM2. SOMAScan of respiratory mucosa analyzed over 1,000 secreted proteins, identifying 162 differentially expressed proteins, including IL-6 and CXCL10, as highly expressed proteins in human influenza infection patients (Marion et al., 2016). SOMAScan was used to diagnose *M. tuberculosis* infection by measuring bacterial antigens (85A, 85B, 85C, CFP2, CFP10, DnaK, GroEL2, GroES, KAD, RplL, and Tpx), with only 85B and CFP10 revealing a significant difference (Russell et al., 2017). Olink uses oligonucleotide antibody pairs containing unique DNA sequences to identify up to 3072 proteins in a sample. Proximity extension through oligonucleotide hybridization creates unique DNA reporter sequences that improve multiplexing and limit antibody cross-reactivity. Brown et al. (2022) investigated the inflammatory response of polymicrobial wound biofilm in an *in vitro* DM foot infection model using Olink proteomics, discovering elevated levels of CDCP1, CXCL1, IL-20, IL-8, MMP-1, TGF-alpha, and VEGFA. Petrera et al. (2021) compared the proteomics of DM, cardiovascular disease, and lung disease patients using DD-MS, DIA-MS, and Olink, measuring 300 proteins using MS techniques and 728 proteins using Olink. The study observed 35 overlapping proteins, highlighting the limitations and benefits of both techniques as well as demonstrating their complementary strengths. Furthermore, the proteins discovered in untargeted MS can be used as targets for immunoproteomics approaches.

### Immunoproteomics

**Figure 2:**
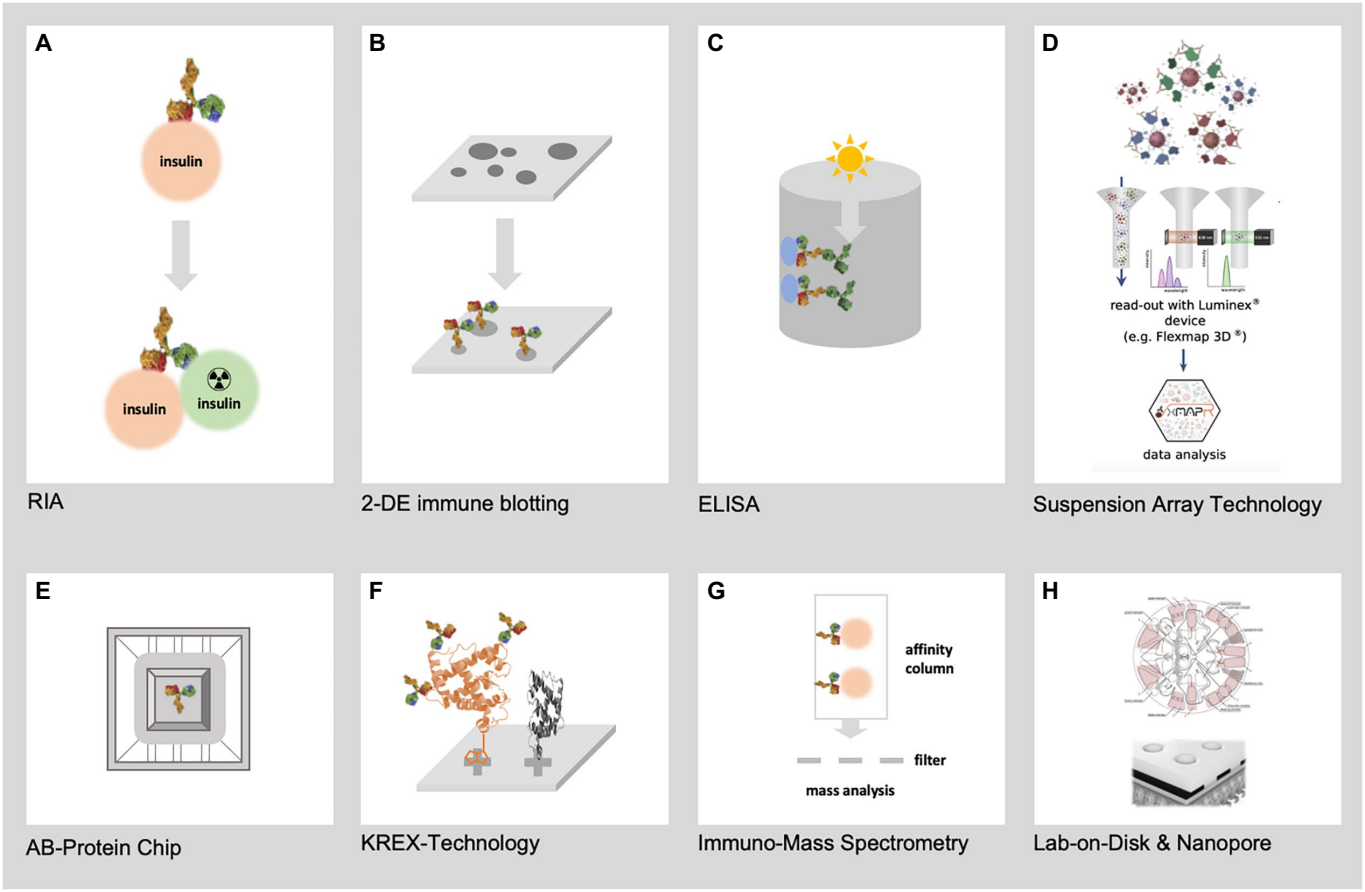
Represents the most relevant techniques for immunoproteomics. **(A)** First immunoproteomics approach (RIA) to detect AB against insulin **(B)** 2-DE immune blotting combining 2-DE electrophoresis of hundreds of native pathogen proteoforms and Western blotting **(C)** Classical ELISA technology using single recombinant proteins **(D)** SAT technology such as FLEXMAP 3D for up to 500 different recombinant proteins **(E)** AB-Protein chips for the detection of thousands of AB against recombinant proteins **(F)** KREX technology for the detection of AB against thousands of correctly folded recombinant proteins **(G)** Mass spectrometry based immunoproteomics using native pathogen proteins **(H)** New lab-on-chip and Nanopore technologies will allow the miniaturization of immunoproteomics in high dimension from recombinant and native pathogen proteins.

Immunoproteomics draws on proteomics to systematic study the adaptive immune system, measuring antigenic peptides and antibodies directed against them. Immunoproteomics refers to a rapidly expanding collection of techniques (Figure 2) for identifying and quantifying antigen proteins and *in silico* methods to understand disease pathogenesis, prognosis, and vaccine development (Fulton et al., 2019). Affinity-based assays that use antibodies coupled to a reporter (florescence, luminescence, radioactivity, or enzymatic activity) remain the gold standard for disease diagnosis. These assays are routinely used to diagnose and classify DM based upon conventional biomarkers, such as HbA1c, fructosamine, adiponectin, glycated albumin, and leptin (Dorcely et al., 2017; Chen and Gerszten, 2020). Enzyme linked immunosorbent assay (ELISA), radioimmune assay (RIA), and Chemiluminescence immunoassay (CLIA) are the most common techniques in immunoproteomics. Perhaps, these assays lack multiplexity, as they can only measure one analyte per reaction and cannot provide an unbiased measurement of all proteins present in the reaction. Recent developments in affinity-based assays have increased the efficiency and spectrum of protein quantification. Multiplexing antigens or antibodies in samples has significantly increased the number of proteins measured in one reaction.

Gel-based immune assays pioneer multiplexing by combining Western blotting for the detection of antigenic proteins and mass spectrometry-based identification. Using Western blotting and transcriptomics improves our understanding of the pathophysiology of infection-induced delayed healing of DM wounds (Ramirez et al., 2018). Chip-based proteome arrays have greatly improved the throughput of proteome-wide antibody screening. Particularly, natively folded, full-length, and completely functional proteins expressed using the KREX technology are used in high-density immunome arrays, including autoimmune, mutant, and epitope screening assays (Poulsen et al., 2020). The groundbreaking technology is widely used to discover novel biomarkers in cancer, autoimmune disorders, and infection biology research (Sumera et al., 2020; Schmidt et al., 2022).

Immunocapture MS, multiple affinity protein profiling (MAPPing), and nucleic acid programmable protein array (NAPPA) are a few of the other novel antigen expression arrays that are recently developed for high throughput host immune response screening and autoimmune analysis (Bian et al., 2016, 2017; Fulton et al., 2019). NAPPA uses cell-free expression systems to directly translate cDNA-encoded bait (target) proteins on glass slides. Anti-tag antibodies co-spotted with cDNA precisely capture *de novo* synthesized proteins. Co-expression of bait and inquiry proteins is captured using the *in vitro* transcription/translation (IVTT) system, allowing efficient and reproducible analysis of protein interactions (Montor et al., 2009; Yu et al., 2015). The query protein is IVTT co-expressed with the bait protein in protein interaction assays to study autoimmune responses, protein–protein interactions, and vaccine development (Arévalo-Pinzón et al., 2018). Using NAPPA, Yu et al. (2015) discovered 10,000 interaction networks of human proteins with *Legionella pneumophila* antigen proteins (SidM and LidA). NAPPA immunoproteomic analysis of *H. pylori* revealed IgG humoral immune response, characterized by a panel of anti-Ggt, -HslU, -NapA, and-CagA antibodies to differentiate gastric cancer patients from healthy control (Song et al., 2021). Similarly, Bian et al. (2016) studied antiviral antibodies in new-onset DM1 using NAPPA protein–protein interactions immunoproteomic profiling arrays, discovering higher antibody responses against EBV in DM1 patients.

Similarly, microsphere suspension array technology (e.g., Luminex FLEXMAP 3D®), which allows for multiplex analysis of up to 500 analytes, is also gaining popularity in immunoproteomics and infection studies. The technology offers flexible options for optimization and multiplexing by binding pathogen antigens with the microspheres to study antibody responses, as previously reported for EBV, papillomavirus, and *M. tuberculosis* (Altuglu et al., 2007; Clifford et al., 2007; Khan et al., 2008). Jiménez-Munguía et al. (2017) studied humoral immune responses against *Pneumococcal pneumonia* to develop a diagnostic array for antigens against infectious pneumonia. *S. aureus* immunoproteomics is performed using suspension array technology (e.g., Luminex® FLEXMAP 3D) to quantify up to 64 antigens across a broad dynamic spectrum (Stentzel et al., 2015). The array was used to examine *S. aureus* IgG profiles in sepsis and non-sepsis patients, revealing a protective signature of eight proteins for the bacteria (Stentzel et al., 2015). Another similar study discovered a panel of eight *S. aureus* proteins that can help early diagnosis of septicemia and could potentially develop *S. aureus* vaccine (Michalik et al., 2020). The authors found that the IgG marker panel against MsrB, FadB, EsxA, Pbp2, FadB, SspB, and SodA proteins could accurately discriminate early *S. aureus* infection (ROC-UAC 0.98). However, these studies did not observe differences in antibody responses through both studies had diabetic and non-diabetic patients infected with *S. aureus* (Stentzel et al., 2015; Michalik et al., 2020). In contrast, a general population study of 996 individuals that tested specific IgG and IgA responses to 79 *S. aureus* antigens found that IgG binding to 66 antigens decreased with increasing BMI and serum glucose concentrations (Meyer et al., 2021). The study observed that smoking, BMI and blood glucose levels moderately influenced the *S. aureus* IgG response (Meyer et al., 2021). Antibody response against naturally occurring 202 glycans, including aminoglysides, glycolipids, ganglio-series, O-linked glycans, and blood group A and B antigens, were observed using FLEXMAP, reporting an association with pathogenesis and progression to DM1 (Tran et al., 2022). Particularly, antibodies against gentamicin and its related structures, G418 and sisomicin, were associated with islet autoimmunity.

To clinically validate the *S. aureus* 4-Antigen (SA4Ag) Vaccine (clinical trial no. NCT02388165), Luminex immunoassays are being used to study humoral immune response against *S. aureus* CP5, CP8, rmClfA, and rMntC proteins (Begier et al., 2017). Furthermore, SA4Ag vaccine efficacy in obese, diabetic, and metabolic syndrome patients was tested using Luminex immunoassays (Scully et al., 2017). Although no significant difference in antibody responses against SA4Ag proteins was observed among different study cohorts, the researchers predicted that an effective *S. aureus* vaccine could enhance neutrophil-mediated bacterial killing and prevent lethal complications in these comorbid populations (Scully et al., 2017). Farnsworth et al. (2015) investigated the humoral immune response to *S. aureus* infections in mice with DM2 and DM1. Total IgG and total IgM levels were determined using whole *S. aureus* extract and selected *S. aureus* intracellular, attachment, and functional proteins (Amd, ClfA, ClfB, FnbA Gmd, IsdA, IsdB, and IsdH). Compared with the controls, infection severity was much worse in both mouse models of diabetes, with DM2 mice generally having lower anti-*S. aureus* IgG response and higher IgM response.

Oh et al. (2018) used Luminex multiplex immunoassay to measure anti-*S. aureus* IgG levels in the serum of DFI patients. The study showed strong prognostic and diagnostic potential of IgG antibodies against *S. aureus* IsdA, IsdH, Amd, Gmd, and Hla antigens in DFI with more than 0.85 ROC-AUC for anti-IsdA and anti-IsdH antibodies. The findings suggest that early detection of *S. aureus* exoproteins using multiplex immune assays improves disease prognosis and holds promise for vaccine development.

## Conclusion

Available literature reveals that the prevalence and severity of microbial infections is higher in diabetic patients. The molecular interactions between infection and host immunity are very complex. Effective preventive strategies, such as vaccination or early detection of diabetic infections, are critical for reducing DM complications and fatalities. The current review discusses the most common DM infections and their prognostic role in DM pathogenesis. *Staphylococcus aureus* is the most contagious and lethal pathogen in SSTIs and septicemia. Both proteomics and immunoproteomics complement each other and have the potential to discover new biomarkers. The lack of reliable infection markers suggests that the diagnostic scope of immunoproteomics, which uses both infection and host marker molecules, should be expanded. Immunoproteomics holds promise for early infection diagnosis, emphasizes the protective function of the adaptive immune system, and promotes further research in producing a clinically successful *S. aureus* vaccine. The above-mentioned latest proteomics and immunoproteomics techniques represent a new milestone in sensitivity, selectivity, and automation analytics. As we can now screen large cohorts for antibodies to pathogens and autoimmune antibodies, a new chapter is being opened in studying the role of antibodies in various diseases. Immunoproteomics techniques can plausibly identify new targets for vaccines and can also be used to conclude mimicry of pathogenic proteins, which has been poorly studied and was not possible on a large scale. However, recent immunoproteomics study of antigenic mimicry of viral and bacterial antigens, as investigated for autoantibody response against blood coagulation proteins (Root-Bernstein et al., 2022), heralds a new era in metabolic disease research. Therefore, the authors believe antibody screening should be a standard part of the future infection-and diabetes-related cohort screening.

## Author contributions

All authors listed have made a substantial, direct, and intellectual contribution to the work and approved it for publication.

## Funding

This work was supported by the Biomedical Research Program at Weill Cornell Medicine in Qatar, a program funded by the Qatar Foundation. This publication was made possible by NPRP-Standard (NPRP-S) 12th Cycle grant # NPRP12S-0318-190392 from the Qatar National Research Fund (a member of Qatar Foundation). The findings herein reflect the work and are solely the responsibility of the authors.

## Conflict of interest

The authors declare that the research was conducted in the absence of any commercial or financial relationships that could be construed as a potential conflict of interest.

## Publisher’s note

All claims expressed in this article are solely those of the authors and do not necessarily represent those of their affiliated organizations, or those of the publisher, the editors and the reviewers. Any product that may be evaluated in this article, or claim that may be made by its manufacturer, is not guaranteed or endorsed by the publisher.
